# Nonequilibrium ion transport in a hybrid battery material

**DOI:** 10.1126/sciadv.aed1629

**Published:** 2026-06-10

**Authors:** John Cattermull, Ben Jagger, Simon J. Cassidy, Shobhan Dhir, Phoebe K. Allan, Mauro Pasta, Andrew L. Goodwin

**Affiliations:** ^1^ Inorganic Chemistry Laboratory, Department of Chemistry, University of Oxford, Oxford OX1 3QR, UK.; ^2^ Department of Materials, University of Oxford, Oxford OX1 3PH, UK.; ^3^ School of Chemistry, University of Birmingham, Birmingham B15 2TT, UK.

## Abstract

Hybrid materials, which combine inorganic and molecular components, often exhibit structural flexibility that enables unusual functional responses. Among them, Prussian blue analogs (PBAs) are a promising class for post–lithium battery technologies. Here, we show that nonequilibrium transformation processes govern the charge-storage mechanism of a PBA electrode, K_2_Mn[Fe(CN)_6_]. Ostensibly, this behavior mirrors that observed in high-rate cycling of conventional cathodes such as LiFePO_4_ yet arises here for fundamentally different reasons–namely, low elastic moduli and cooperative distortions inherent to the hybrid framework. Using operando x-ray absorption spectroscopy with Metropolis matrix factorization and x-ray diffraction, we show that framework flexibility limits transport kinetics and promotes collective, metastable pathways. Our results not only highlight various directions for PBA cathode optimization but also suggest a broader relevance of nonequilibrium mechanisms for mass transport in hybrid materials beyond PBAs alone.

## Teaser

Mechanistic insights uncover how the flexibility of a hybrid framework controls ion transport, revealing pathways to optimization.

## INTRODUCTION

Electrochemical processes, such as those occurring within electrode materials during battery cycling, often couple strongly to phase transformations (*1*). The kinetics of such processes then involve a complex interplay among many competing factors, including charge carrier mobilities (*2*, *3*), elastic stress propagation rates (*4*), and the chemical potential gradients that develop at phase boundaries (*5*). A consequence of this complexity is the emergence of nonequilibrium thermodynamics in battery materials, whereby transformation mechanisms can vary with cycling rate and/or particle size. One particularly well-known example is that of LiFePO_4_, which transforms via a thermodynamically forbidden solid solution under high rates of cycling (*6*–*8*). Another is that of Li(Ni,Mn,Co)O_2_ (NMC), where the conventional theory of single-phase behavior in Li compositions of 0.5 < *x* < 1 was recently challenged by direct observation of phase separation using advanced operando characterization (*9*). In that system, state of charge heterogeneities arise due to composition-dependent reaction rates (*10*–*12*). The importance of understanding nonequilibrium behavior is a crucial step to enable informed materials optimization through the tuning of particle size, morphology, and disorder (*13*–*15*). The relevance of these considerations to battery technologies based on hybrid chemistries remains largely unknown (*16*).

Prussian blue analogs (PBAs) are an increasingly prominent family of cathode materials for both Na- and K-ion batteries, yet their electrochemical transformation mechanisms are remarkably poorly understood (*17*). From an application perspective, the key materials make use of cheap, abundant elements with the redox of Mn or Fe coupled to the reversible (de)insertion of Na- or K-ions, which are key criteria for next-generation battery materials to ease the dependence on critical minerals (*18*). The broader family of PBAs includes a large variety of systems (*19*–*21*). Common to all is an open framework structure composed of molecular M′─CN─M linkages, which impart a flexibility not observed in conventional oxide or polyanion cathode materials (*22*). More generally, the wider range of bonding interactions in hybrid inorganic materials is crucial to their remarkable performance in various applications, including perovskite optoelectronics (*23*). The PBA structure is made up of a simple cubic lattice decorated by alternating M^*m*+^ and [M′(CN)_6_]^*n*−^ ions with Na- or K-ions occupying the pore-space within. [M′(CN)_6_]^*n*−^ vacancies (up to one-third per formula unit) are a common feature of PBA chemistry; these dictate the number of counterions incorporated within the cubic framework cavities to balance charge. It is the softness of the molecular interactions in PBAs that means the materials can be synthesized at room temperature from aqueous solution (*24*, *25*); but this softness also promotes a variety of low-energy distortions (*26*, *27*).

It has become increasingly apparent that the structural distortions in PBAs are intimately connected to their electrochemistry (*28*, *29*). Structural distortions are a particular characteristic of low-vacancy PBAs, due to increased connectivity and higher alkali-metal cation content (*30*). Such low-vacancy PBAs promise higher theoretical capacities but cycle via multiple phases and thus have a reduced rate capability (*31*, *32*). In the pristine state of low-vacancy PBAs, distortions are dominated by cooperative displacements of the alkali-metal cation (*33*), and when the cathode is fully charged, distortions arise from framework strain (*31*). By contrast, high-vacancy PBAs show no cooperative distortions: Their disordered cubic structure cycles via a solid solution with high rate capability (*34*). From a design perspective, the suppression of distortions in low-vacancy PBAs has been an obvious target because it would allow high rate capability while preserving high capacities (*28*). However, without a mechanistic understanding of how the phase transformations of low-vacancy PBAs couple to the electrochemistry, it is difficult to strategically target higher performing PBA electrode chemistries. Note that simulating PBAs ab initio is prohibitively challenging due to large unit cells, high degrees of vacancies and disorder, as well as a landscape of low-energy distortions shown to fluctuate with moderate changes in temperature (*35*, *36*). Careful experimental study of these mechanisms is therefore key to unraveling the complex electrochemical behavior and its interplay with composition and structure.

To address this fundamental problem, we study here the K-ion cathode, K_2_Mn[Fe(CN)_6_], as a model system. Our choice of material was influenced by the following considerations. First, it is this PBA that can be prepared as an anhydrous, vacancy-free material, with well-separated monolithic particles (*37*). This is important if we are to investigate electrochemical cycling effects independent from complications of varied particle morphology and water-based high-voltage degradation. Second, among PBAs, its structure and associated framework distortions are particularly well characterized (*27*, *33*). Third, K_2_Mn[Fe(CN)_6_] has a high theoretical capacity of 155 mA·hour g^−1^ due to low vacancy content, which, combined with the high operating voltage of the Mn^3+^/Mn^2+^ couple (close to 4 V versus K^+^/K), enables K-ion batteries with a specific energy density competitive with state-of-the-art LiFePO_4_/graphite cells (*37*). We use operando x-ray absorption spectroscopy (XAS) and x-ray diffraction (XRD) to study the phase transformation mechanisms of this PBA electrode during a single charge/discharge cycle. We will come to show that its electrochemistry is governed by multiphase behavior due to subtle structural changes and that the two different structural transformation mechanisms that occur at high and low potassium contents are nonequilibrium in their nature. The underlying cause of this nonequilibrium behavior is the flexibility of the PBA framework, which acts to couple ion transport with framework deformations and inhibits domain-wall motion. These considerations are widespread among hybrid materials, and hence our results are likely to be relevant to challenges of ion transport in the broader family of hybrid systems.

## RESULTS

### Charge behavior

**Fig. 1.**
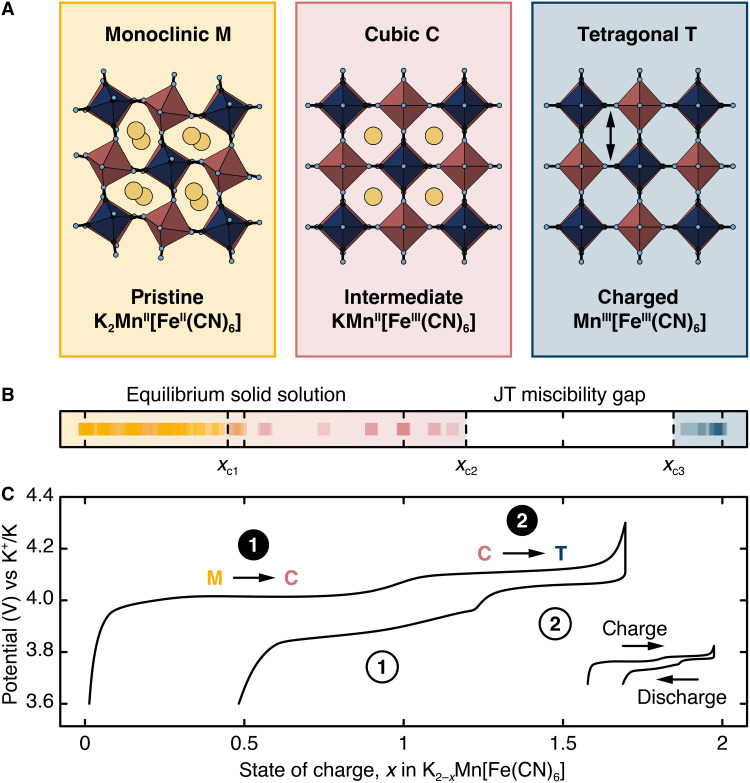
Structures of K_2−*x*_Mn[Fe(CN)_6_] via chemical and electrochemical preparation. (**A**) Schematic structural representation of the three PBA phases present during electrochemical cycling. In the case of JT-distorted Mn[Fe(CN)_6_], arrows are included to represent the anisotropy due to JT-active Mn(III). K is shown as amber spheres, FeC_6_ is shown as dark blue octahedra with black vertices, and MnN_6_ is shown as brown octahedra with light blue vertices. (**B**) Samples of composition K_2−*x*_M[M′(CN)_6_]*_y_* [reported in refs. (*30*, *39*, *49*)] are plotted as a function of *x* and colored according their crystallographic symmetry. A dashed line at *x* ∼ 0.45 denotes a threshold value, *x*_*c*1_, separating monoclinic and cubic phases. The apparent JT miscibility gap for intermediate composition of JT-active metal is similarly highlighted with *x*_*c*2_ and *x*_*c*3_ as the solubility limits of the cubic phase (*x* ∼ 1.15) and tetragonal phase (*x* ∼ 1.85), respectively. (**C**) Pseudo-OCV charge-discharge profile of the K_2_Mn[Fe(CN)_6_]/K half-cell from the galvanostatic intermittent titration technique. Regions **1** and **2** are highlighted to represent the monoclinic to cubic transition and cubic to tetragonal transition, respectively, and are discussed in the main text. Filled circles denote processes during charge, and open circles denote processes during discharge.

On charging, the K_2_Mn[Fe(CN)_6_] cathode transforms via two processes, with the pristine and fully charged states having different structures, and each different again to a third partially-charged intermediate phase (Fig. 1A) (*31*). The pristine K_2_Mn[Fe(CN)_6_] has a monoclinic structure owing to the K-ion slide distortion—a cooperative off-centering of K^+^ ions within the cavities toward an edge of the surrounding anionic PBA framework that couples to octahedral tilts (*33*). Under (equilibrium) chemical control, low-vacancy compositions such as ours can prepared with K-ion content as low as 1.6 per formula unit while still crystallizing as a single-phase monoclinic structure (*38*). For lower K-ion contents, these PBAs adopt a cubic structure instead, as a result of the weakened driving force for cooperative K-ion off-centering (Fig. 1B) (*30*). The fully charged K_0_Mn[Fe(CN)_6_] contains Jahn-Teller (JT) active Mn(III), which induces a tetragonal distortion of the framework through a cooperative JT effect. The associated strain is why JT solubility is so poor in the cubic phase of low-vacancy PBAs; for example, in the system Mn^II^*_x_*Cu^II^_1–*x*_[Pt(CN)_6_], a miscibility gap is observed for 0.15 < *x* < 0.85 (Fig. 1B) (*39*). Hence—from an equilibrium perspective—one expects conversion between the three phases on charging K_2−*x*_Mn[Fe(CN)_6_] to occur first by a continuous solid solution phase change from monoclinic to cubic symmetries at some critical K-ion content x≃0.45, followed by a second phase change from a cubic phase with x≃1.15 to increasing fractions of a tetragonal phase with x≃1.85.

In practice, as K_2_Mn[Fe(CN)_6_] is cycled, two voltage plateaus are observed, each contributing around half of the capacity and each corresponding to the (de)insertion of 1 mol of K^+^ ions from the PBA and an associated phase transition (Fig. 1C) (*31*). Referring to Fig. 1C, in region **1**, the pristine (K_2_Mn[Fe(CN)_6_]) phase begins converting to the intermediate (K_1_Mn[Fe(CN)_6_]) phase almost instantly with charging, without the expected solid-solution behavior. The immediate onset of this transition is evidence of nonequilibrium behavior because chemical preparation of the PBA can produce a range of single-phase compositions with K-ion concentrations between 0 < *x* < 1 (Fig. 1B), implying that a solid-solution mechanism should be possible. In region **2**, the conversion of K_1_Mn[Fe(CN)_6_] → K_0_Mn[Fe(CN)_6_] is coupled with the emergence of a cooperative JT distortion from the presence of Mn^3+^. With this in mind, it is expected that K_2−*x*_Mn[Fe(CN)_6_] should phase separate for intermediate values of *x*. Although these two transformations have been observed experimentally in previous studies (*28*, *31*, *36*, *40*, *41*), little is known about the transformation mechanisms themselves. In particular, there is no quantitative understanding of the population of each phase for a given state of charge nor that of any compositional or structural changes within the phases themselves. We anticipate that the mechanisms of the transformations will be central to rationalizing the performance of the cathode material.

**Fig. 2.**
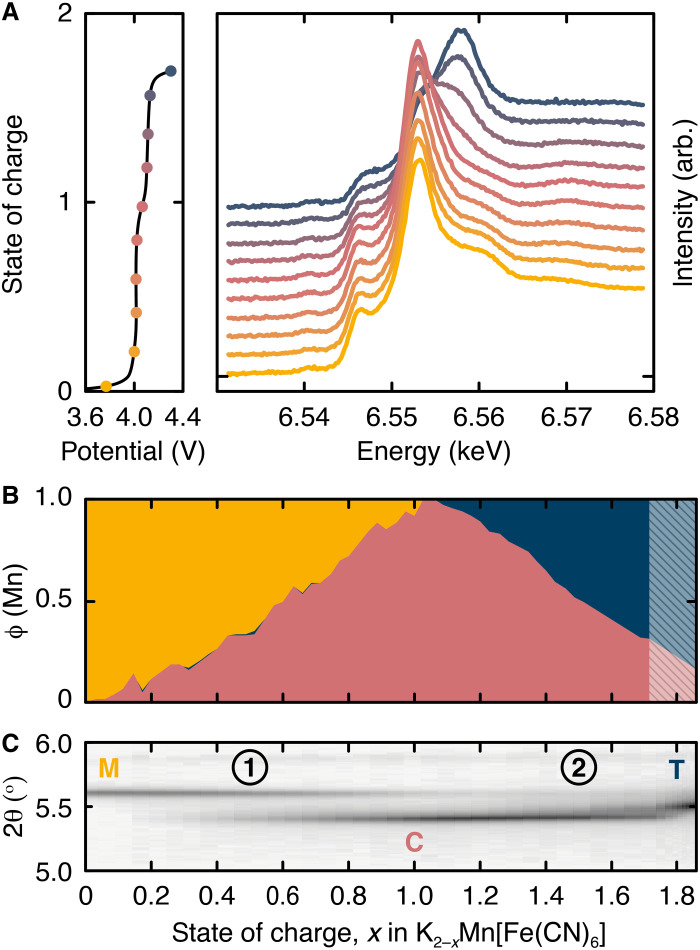
Operando characterization of the K_2−*x*_Mn[Fe(CN)_6_] cathode. (**A**) Normalized XAS profile for the Mn K-edge with selected curves offset vertically by a constant amount and colored by state of charge to map onto the simplified voltage profile alongside. arb., arbitrary units. (**B**) MMF phase fractions for the fixed MMF refinement of the XAS data. The pristine spectrum is in amber, the intermediate spectrum is in pink, and the Mn(III) component is in dark blue. The shaded region is extrapolated to match up with the XRD plot in (C), where a constant voltage hold at 4.3 V drove the state of charge to a maximum of *x* = 1.84. (**C**) Film plot for the region around the (200) XRD reflection of the parent cubic structure to show the structural phase changes on cycling. The monoclinic phase appears to monotonically convert to the larger cubic phase before finally converting to the smaller tetragonal phase late in charge.

Our starting point in linking electrochemistry to structural transformations was to use operando XAS to track the changes in oxidation state during cycling via the K-edge energy profiles of Fe and Mn. The Fe K-edge shift turns out to be relatively insensitive to charge state as a consequence of covalency in the Fe─CN interaction (fig. S4B). By contrast, the Mn K-edge profile tracks the oxidation state of the PBA and is even indirectly sensitive to Fe charge state, due to its sensitivity to local structure, with a clear change in profile in the first charge plateau (Fig. 2A). The clearly defined voltage plateaux associated with the redox of each metal indicate that the XAS data ought to be separable into components of each charge state species—namely, pristine, fully charged, and a partially charged intermediate. For that reason, we used Metropolis matrix factorization (MMF) to analyze the XAS spectra for Mn (*42*). MMF refinement of the operando XAS data using three components confirmed that the Fe^2→3+^ redox couple is active in the first charge plateau and Mn^2→3+^ in the second charge plateau (Fig. 2B). The XAS/MMF analysis also enables accurate normalization of capacity by state of charge, represented as units of *x* in K_2−*x*_Mn[Fe(CN)_6_]. A more detailed discussion of the MMF fitting is given in the Supplementary Materials. The link between capacity and state of charge so established then allows normalization of operando XRD data collected under similar conditions (Fig. 2C).

### First charge plateau

**Fig. 3.**
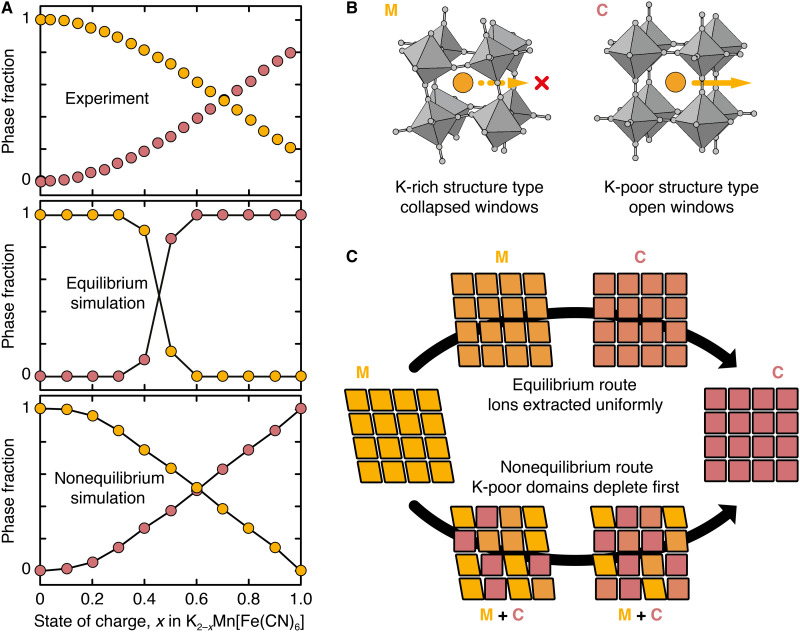
Operando XRD analysis of the first charge plateau. (**A**) XRD refinement phase fractions as compared to the predicted phases from equilibrium and nonequilibrium simulations. (**B**) Schematic structural representation of the effect of opening the framework windows on K-ion transport when the monoclinic phase transforms into the cubic phase. (**C**) Evolution of coherent scattering domains with K-ion extraction in the case of equilibrium and nonequilibrium transformations. On the basis of the simulations, we interpret the experimental phase fraction evolution to represent the nonequilibrium route here.

To interpret our operando XRD data, we turn our attention first to the charge region **1**, where the pristine phase begins to convert into an intermediate phase early on in the charging process (Fig. 2C). Refinement of the XRD patterns allows the phase fractions to be expressed as a function of state of charge, as summarized in Fig. 3A (refinement method is discussed in the Supplementary Materials). We see a two-phase reaction, which indicates that K-ions are not being removed homogeneously from the PBA of variable composition K_2−*x*_Mn[Fe(CN)_6_] (0 < *x* < 1)—i.e., via a solid-solution mechanism. The two phases form in the same way at faster cycling rates and persist even after relaxation for several hours (fig. S9). From an equilibrium perspective, the pristine monoclinic phase is stable on depotassiation until *x* ∼ 0.45 before its conversion to the intermediate cubic phase (Fig. 1B), and hence one would expect onset of phase transformation only at the relevant charge state close to this stoichiometry. The experimental departure from this picture suggests K-ion extraction is under kinetic control during electrochemical K-ion extraction, with a barrier to extraction that is dependent on loading, *x*. Our interpretation here is based on similar behavior reported for Ni-rich NMC cathodes. In the case of NMC, concentration-dependent diffusivity produces a “core-shell” structure of lithium-poor peripheries and lithium-rich cores in single-particle cathodes at the beginning of charge, giving rise to a closely related operando XRD profile (*12*). What would drive such a nonequilibrium pathway in PBAs? In the highly potassiated state, the PBA framework is collapsed to maximize its interaction with the K^+^ ions; the degree of collapse scales with K-ion concentration, pinning the K-ions less strongly as the K-ion composition is reduced (*27*, *33*). The picture that emerges is one where K-ion mobility increases for higher values of *x*, meaning that, as K-ions are removed from crystallites of K_2_Mn[Fe(CN)_6_], subsequent removal is favored in locally depleted regions where the framework is opened up, increasing the area of the framework windows by ∼50% (Fig. 3B). Previously, we showed that the K-ion composition affects the effective diffusivity of this exact same PBA sample and cell composition (*37*). A simple model that incorporates this composition-dependent mobility captures unexpectedly well the key phase behavior observed experimentally (Fig. 3A) (see the Supplementary Materials for further discussion). In addition, the nonlinear phase conversion for *x* < 0.25 would be consistent with the apparent change in slope visible early in the first charge plateau in Fig. 1C. Thus, our experimental data support a model in which phase transformation occurs heterogeneously throughout PBA crystallites (Fig. 3C).

This behavior in K_2_Mn[Fe(CN)_6_] contrasts with that of other commonly studied cathode chemistries. In LiFePO_4_, for example, there is a strong thermodynamic driving force for phase separation such that intermediate compositions Li*_x_*FePO_4_ are unstable (*43*). Lithium mobilities in that system are clearly much higher in the absence of an applied potential than the potassium mobilities in PBAs, such that kinetically obtained intermediate compositions Li*_x_*FePO_4_ are able to relax quickly to LiFePO_4_/FePO_4_ mixtures (e.g., on an ∼10-s timescale after charging at 10 *C*) (*7*). No such relaxation to single-phase equilibrium compositions is possible for our PBA samples on the experimental timescale we probe. Thus, despite its apparent simple biphasic plateau, K_2_Mn[Fe(CN)_6_] is actually more similar to Ni-rich NMC and almost in direct contrast with LiFePO_4_, in that the combination of slow K-ion mobility, relatively weak electrostatics, and K-ion composition–dependent kinetics changes what should be a solid-solution transformation into a two-phase process.

### Second charge plateau

**Fig. 4.**
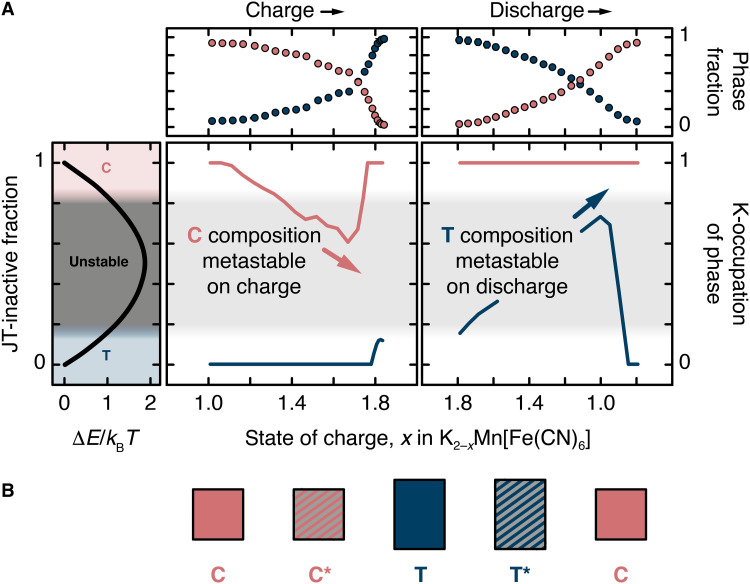
Operando XRD analysis of the high voltage plateau. (**A**) XRD refinement phase fractions are plotted as a function of state of charge. The strain map from ref. (*39*) is used to visualize the strain of the incumbent phase (gray region) based on necessary change in K-ion occupancy calculated from *x*. The pink and dark blue lines are the K-occupation of the intermediate and fully charged phases, respectively. *k*_B_, Boltzmann constant. (**B**) The strain present in the PBA is illustrated schematically. On charge, the strain in the intermediate phase, labeled C, builds up with little change in phase fraction before relief of that strain by accelerated phase transformation. On discharge, the reverse occurs, i.e., the strain builds up in the fully charged phase, labeled T, with slow phase conversion, before faster phase conversion relieves the strain.

We turn our attention now to region **2**, where cubic K_1_Mn[Fe(CN)_6_] is converted into tetragonal K_0_Mn[Fe(CN)_6_] (Fig. 1). This composition range differs from the first in that, under equilibrium conditions, no intermediate phases have been isolated—a consequence of the strain-driven JT insolubility discussed above. Our XAS data showed a clear phase change relating to Mn, where one peak is consumed as another grows in at higher energy with a clear isosbestic point (Fig. 2, A and B). In addition, the extended x-ray absorption fine structure (EXAFS) analysis confirms that the Mn-octahedra are distorted on oxidation to form two distinct bond lengths (fig. S4). The phase change is also visible in the operando XRD patterns as a third phase appears (Fig. 2C). Constrained refinements of the XRD traces allowed us to quantify the relative fractions of cubic and tetragonal phases, but we found that the quantities of those phases were not sufficient to account for the known charge states (Fig. 4). For example, at a state of charge of *x* = 1.50, only 20% of the material has converted to the fully charged phase, when, from the electrochemistry, one would expect 50%. The accompanying decrease in lattice parameter is evidence for change of composition within the two phases during charge (fig. S11), by accommodation of distorted MnN_6_ octahedra within the intermediate phase, also clearly present in the Mn EXAFS (fig. S4). It is only near the very end of the charge plateau—and on application of a final constant voltage hold—that the fully charged phase suddenly emerges in appreciable quantities (Fig. 4). On discharge, a clear hysteresis in phase fraction evolution is observed, with the fully charged tetragonal phase now dominant longer than anticipated. Together, these observations are consistent with a substantial kinetic barrier to interconversion between the two phases (Fig. 4). The sluggish transformation kinetics mean that the dominant phase present must accommodate strain associated with K-ion concentration changes and accompanying Mn redox beyond its equilibrium stability field (Fig. 4).

Conventional multiphase cathode materials rely on high elastic constants to drive the phase boundary wave on charge and discharge (*4*). However, the softer bonding interactions in the PBA framework and stronger elastic compliance allow strain to build up in the framework without inducing phase change (*44*), reducing the driving force for phase boundary wave propagation. Any delay in phase transformation fundamentally limits the rate capability of a material (*45*), which is likely why PBAs exhibit low charge storage capacities at high cycling rates (*36*). In other systems—e.g., LiFePO_4_—wave propagation can accommodate the formation of metastable solid-solution phases when cycling rates are faster than phase transformation process. That is to say, the coherency strain in ceramics with higher elastic constants aids diffusion (*1*), but in more flexible materials such as PBAs, the flexibility hampers ion diffusion by slowing the phase transformation. A corollary of this argument is that the lack of phase transformations in high-vacancy PBAs therefore explains the markedly superior rate capability found in those systems (*34*).

## DISCUSSION

Our key result shows how nonequilibrium transformation mechanisms extend from conventional cathode materials to hybrid materials such as PBAs, albeit for different microscopic reasons. Our study also has the practical outcome of identifying potential strategies for optimization of electrochemical performance in K-ion PBAs. For example, improvement to the kinetics in the first charge step might be achieved by lowering the initial K-ion concentration (*27*), exchanging Mn for smaller transition metals (*33*), or pinning open the framework with a low level of Cs^+^ doping—all of which would stabilize the cubic phase to higher K-ion concentrations (*46*). These potential strategies would have to be tensioned against the associated reduction in specific energy density of the material in each case. The more conventional strategy of targeting smaller particle sizes (*47*) increases the relative number of available K-ion extraction sites but does so while actually increasing the fraction of K-ions with high kinetic barriers to extraction. This same strategy would be advantageous, however, for the second charge step, accelerating the phase transformation from cubic to tetragonal and potentially improving reversibility. An alternative consideration to varying particle size is to use different hexacyanometallate vacancy correlations to optimize the interplay between vacancy fraction and phase stability (*48*). The ultimate goal here is to stabilize the undistorted intermediate cubic phase for all K-ion compositions at as low a vacancy fraction as possible. These dual directions of controlling particle size and defect engineering will alter the nonequilibrium behavior of PBAs, and we intend to explore both aspects in follow-up studies.

The microscopic ingredients responsible for nonequilibrium ion transport in K_2_Mn[Fe(CN)_6_] are relevant to all PBAs and to hybrid materials more generally. For example, distortion mechanisms and their coupling to mobile ion occupancies obey universal trends among PBAs (*30*, *49*) and thus will affect ion-transport mechanisms of other important PBA electrode materials (e.g., Na_2_Fe[Fe(CN)_6_]) (*50*). Low-vacancy PBAs with framework distortions in the pristine state, irrespective of transition metal or mobile ion composition, show biphasic behavior on charging where solid solution is the equilibrium state (*28*, *31*, *41*, *51*, *52*). In metal-organic frameworks—which generally share the elastic compliance of PBAs (*53*)—sluggish transformation kinetics associated with guest-driven phase transformations [e.g., in MIL-53 (*54*) or DUT-49 (*55*)] might now be viewed through the same lens used here to rationalize the second charge mechanism of K_2−*x*_Mn[Fe(CN)_6_]. The same is likely true in hybrid perovskite photovoltaics, where both ion diffusion and strain localization are key to chemical stability and long carrier lifetimes, respectively (*56*, *57*). Whatever the particular family, our results highlight that the unique and general features of hybrid materials—their diversity of structural distortions and anomalous mechanics—will be important design tools not only for equilibrium properties [now routinely explored (*23*, *58*, *59*, *60*, *61*)] but also for controlling the mechanism of functional response away from equilibrium.

## MATERIALS AND METHODS

### Synthesis

Our K_2_Mn[Fe(CN)_6_] sample was synthesized using the procedure described in ref. (*37*). Reagent salts, MnSO_4_ (Sigma-Aldrich, 0.5 mmol) and K_4_Fe(CN)_6_ (Sigma-Aldrich, 0.5 mmol) were dissolved in separate aqueous solutions of potassium citrate (Sigma-Aldrich, 1 M, 50 ml). These solutions were added simultaneously, dropwise (2 ml min^−1^), to a stirring round-+bottom flask containing a 100-ml solution of 1 M potassium citrate at 20°C to ensure that the concentration of both reagents in the reaction flask remained consistent throughout. The mixture was stirred for 24 hours before the precipitate was isolated by centrifugation and washed with a 50:50 water/ethanol mixture to prevent the solid from dispersing. The solid was dried in air at 70°C overnight and then under vacuum at 70°C. Phase purity and structure were confirmed by XRD (fig. S1A). Rietveld refinement gave 0.942(4) occupancy of the K-site (table S1), and inductively coupled plasma mass spectrometry (ICP-MS) was used to determine vacancy content by the mass ratio of Fe:Mn of 0.981(7).

### Scanning electron microscopy

Scanning electron microscopy (SEM) was used to confirm that the target particle size, morphology, and dispersion had been achieved (fig. S1, B and C). Particles were dispersed in acetone and then mounted on an aluminum stub. These particles were imaged using a Zeiss Merlin SEM equipped with a field-emission gun, operated at an accelerating voltage of 3 or 10 kV and a probe current of 100 pA.

### Electrode preparation and cell assembly

Target electrode loadings were determined on the basis of a compromise between achieving representative electrochemistry while being able to attain sufficient signal-to-noise from the various operando x-ray techniques. The PBA electrodes were prepared using a slurry casting method of a composite in the mass ratio of 7:2:1 [active material:Super P carbon (TIMCAL):polyvinylidene difluoride (PVDF) binder] (Sigma-Aldrich) using the *N*-methylpyrrolidone (NMP) solvent (99.5% anhydrous, Sigma-Aldrich). The electrodes were cast a onto carbon-coated aluminum current collector. All electrodes were initially dried in air at room temperature for 12 hours followed by vacuum drying at 100°C for 24 hours. The PBA electrode was lightly calendared to ensure electrical contact.

Potassium bis(fluorosulfonyl)imide (KFSI; 99.9%, Solvionic) was dried under vacuum at 100°C for at least 48 hours, and triethyl phosphate (TEP; 99.8%+, Sigma-Aldrich) was dried over potassium metal strips for at least 1 week. The electrolyte was made as a 2.5 M KFSI solution in TEP and then tested for water content by Karl Fischer titration and recorded to be below 5 parts per million (ppm). The K metal (98%, Sima-Aldrich) was melted under Ar atmosphere, and the impurity layer was removed followed by quenching in mineral oil and storing in anhydrous hexane (95%, Sigma-Aldrich).

The electrodes were assembled in CR2032 coin cells for electrochemical testing in the following way (fig. S2). First, a 1-mm-thick, 10-mm-diameter K metal reference electrode was punched and cleaned by removing the dull surface layer to leave a shiny surface. The K metal electrode was then fixed to a stainless steel current collector, which was placed on top of a spring in the cell “top” with a polypropylene (PP) gasket. An electrolyte-soaked glass microfiber (GF/F grade, Whatman) separator was immediately placed on top of the anode. The PBA electrode was placed on top of the separator, and an aluminum-coated “base” was used due to the high operational voltages. The cell was flooded with electrolyte such that, upon crimping, it leaked excess.

### In-house electrochemical testing

All in-house electrochemical tests were carried out using a Biologic VMP3 battery cycler in a cycling oven set to 30°C. Once assembled, cells were connected up and allowed to rest for 12 hours under open-circuit voltage (OCV), which was typically in the region of 2.9 to 3.2 V.

The first cycle was performed at a rate of *C*/20, where *C* is the number of (dis)charges per hour based on the theoretical capacity (*C* = 155 mA g^−1^). On application of positive current, the cell quickly reaches the first charge plateau at ∼4.08 V before rising to a second plateau at ∼4.16 V and eventually reaching the 4.3 V cutoff (Fig. 1B). On discharge, again the capacity is dominated by two plateaus. Discharge capacity for the cathode active materials is ∼90 mA·hour g^−1^, which is typical for large-particle, low-vacancy K_2_Mn[Fe(CN)_6_] (*35*). To generate the plot in Fig. 1C, a pseudo-OCV was achieved using the galvanostatic intermittent titration technique. A 15-min pulse at current equivalent to *C*/20 cycling was applied followed by a 2-hour relaxation.

### Operando cell

Coin cells were modified for operando experiments to achieve beam transmission through the cell (fig. S2). The stainless steel current collector had a 2.8-mm hole drilled, and a 3.2-mm hole was made in the K metal anode.

For XAS, the coin cell casings were drilled with a 5-mm hole and sealed with Kapton tape to achieve transmission of the lower-energy x-rays used for XAS and ensure that no other Fe-containing components interacted with the beam. Galvanostatic cycling was carried out using an Ivium Octostat200. The cell was cycled at a rate of *C*/20. A small overpotential was observed for the first charge plateau, which could be due to some interaction of the x-rays with the components of the cell or the modified cell setup itself; however, the profile of the charge/discharge curves were otherwise representative.

For XRD, the coin cell casings were instead laser thinned to 50 μm as the x-ray beam was of sufficiently high energy to penetrate the casing. Two cells were measured under galvanostatic cycling: using the same protocol to the XAS cell with an additional constant voltage hold at 4.3 V until the current density dropped to 1 mA g^−1^ (∼*C*/150) to drive the charge further to completion and isolate the fully charged phase as far as possible.

A third cell was measured under pulse/relaxation conditions in its second cycle. The cell was initially cycled in-house for one full formation cycle. After ∼6 hours, the second cycle was started on the beamline. A 2-hour rest followed by a 30-min pulse at *C*/10 was used to test the stability of the separate phases under both faster cycling conditions and relaxation (i.e., no current).

### XAS experiment details

XAS measurements were carried out on the B18 beamline at the Diamond Light Source (UK). The technique was used to probe the oxidation state and local environment of the transition metals, Mn and Fe in the PBA electrode, by measuring the absorption at the corresponding K-edges. Data were collected in transmission mode, by continuous scanning from 6339 keV up to 7662 keV in ∼0.22-keV intervals over 8 min using Fe metal foil for energy calibration of each scan.

Pellets of K_4_Fe(CN)_6_, K_3_Fe(CN)_6_, and K_2_Mn[Fe(CN)_6_] powder were made with cellulose and measured to compare with the pristine electrode measured in the uncycled coin cell (fig. S3). X-ray absorption data were aligned using Athena, part of the Demeter software package (*62*). Fourier transforms of the EXAFS data were made in the *k*-range of 1.9 to 7.65 Å^−1^.

### Synchrotron XRD experiment details

Synchrotron XRD measurements were carried out on the I11 beamline at the Diamond Light Source operating with an x-ray wavelength of 0.493482 Å. The position-sensitive detector was used to measure diffraction of the modified coin cell in transmission mode. Diffraction patterns were summed from two data collections of 2 min each collected at angles 0.25° apart to account for gaps in detector coverage. In a similar protocol to that used for the XAS measurements, a powder sample of K_2_Mn[Fe(CN)_6_] was measured as well as a pristine electrode to confirm that the structure was preserved. A bespoke cell holder was used on a motorized *xyz* sample stage, which allowed measurements in pre-set positions of each cell via small holes (7 mm in diameter), enabling the continuous sequential measurements of all the positions.
