# Supplementary material for: Nonequilibrium ion transport in a hybrid battery material
Source: Sci Adv. 2026 Jun 10;12(24):eaed1629. doi: 10.1126/sciadv.aed1629 (PMC13251832; doi:10.1126/sciadv.aed1629)
Supplement: Supplementary file 1 — Supplementary Text Figs. S1 to S11 Table S1 Legends for data S1 to S3 [file sciadv.aed1629_sm.pdf]

Science Advances
AAAS

Supplementary Materials for

Nonequilibrium ion transport in a hybrid battery material

John Cattermull *et al.*

Corresponding author: Mauro Pasta, mauro.pasta@materials.ox.ac.uk;
Andrew L. Goodwin, andrew.goodwin@chem.ox.ac.uk

Sci. Adv. **12**, eaed1629 (2026)
DOI: 10.1126/sciadv.aed1629

The PDF file includes:

Supplementary Text
Figs. S1 to S11
Table S1
Legends for data S1 to S3

Other Supplementary Material for this manuscript includes the following:

Data S1 to S3

Supplementary Text

XAS/MMF discussion

A pelletised sample of $K_2Mn[Fe(CN)_6]$ was initially assessed to confirm neither transition metal had been oxidised during any processes in cell assembly and rest. The Fe K-edge spectra matched the spectrum measured for a fresh sample of $K_4Fe(CN)_6$ precursor suggesting the PBA contains only Fe^{2+}. The Mn^{2+} is more stable with respect to oxidation and Mn^{3+} has a clearly identifiable XAS signature not present here, so we judge our material to be $K_2Mn^{II}[Fe^{II}(CN)_6]$ [Fig. S3].

During the first charge plateau, the profile of the Mn XAS changes but the K-edge position remains at 6551.3 eV, whereas the Fe K-edge position gradually increases from 7128.3 to 7128.9 eV. XAS is notoriously insensitive to changes to t_{2g} electrons of metals in the presence of a pi-accepting ligands (*41*). The small change in K-edge position for the redox couple Fe^{3+}/Fe^{2+} is therefore interpreted as a reduction in pi-backbonding to the CN-ligand, which mitigates any effect of oxidising Fe(II) to Fe(III). The first charge plateau is interpreted as oxidation of Fe: $K_2Mn^{II}[Fe^{II}(CN)_6] \rightarrow KMn^{II}[Fe^{III}(CN)_6]$.

During the second charge plateau the Mn XAS changes dramatically in a biphasic reaction. A new peak at higher energy forms as the other peak is consumed with an isosbestic point at 6556.2 eV. This phase change is interpreted as $KMn^{II}[Fe^{III}(CN)_6] \rightarrow Mn^{III}[Fe^{III}(CN)_6]$ where Mn(III) is JT active. The Fe K-edge continues to increase in energy during the second charge plateau as a response to the oxidation of neighbouring Mn and its resultant change in ligand field.

The X-ray absorption spectra were normalised by fitting a straight line to the pre-edge region so $I_{\text{pre-edge}} \sim 0$ and a quadratic to the post-edge region so $I_{\text{post-edge}} \sim 1$. Once normalised, the Mn spectra were fitted using the hybrid multivariate analysis algorithm Metropolis Matrix Factorisation (MMF) developed in Ref. 42. Metropolis Monte Carlo minimisation uses non-negative matrix factorisation to generate physically meaningful components of the XAS spectra in a way that more conventional principal component analysis cannot, without complex parameterisation. Therefore the components generated by the MMF analysis can be directly interpreted as the XAS profile for a specific state of charge.

Fixing the spectrum of the pristine electrode as one component ($K_2Mn^{II}[Fe^{II}(CN)_6]$), a further two components were allowed to refine freely in the MMF analysis. The MMF quickly minimised

with an excellent fit-to-data (R_{wp} = 12.61%) with the accompanying phase fractions and components shown in Supplementary Tables. This fit-to-data for three components was much better than when only two were used (R_{wp} = 15.22%), justifying the inclusion of the extra component.

The phase which emerges after half way through full charge is interpreted as containing Mn(III) and the transition between the other two phases as oxidation of the Fe during the first charge. The pristine phase is consumed more slowly early on charging of the cell than it is for the rest of the plateau. From a charge capacity of 12 mA h g^{-1} onwards, the phase fraction of the pristine components decreases linearly with capacity. Since the XAS spectrum is a direct probe of the electronic environment of Mn (accounting for both oxidation state and local structure), the MMF result can be used to determine state of charge (SOC) of the PBA cathode in a more accurate way than charge capacity, whilst also giving information about where the charge is coming from in the PBA cathode. Extrapolation of the linear region of the pristine phase fractions in Fig. S5 indicates that the first 7.5 mA h g^{-1} of charge can be described as non-PBA processes. The scope of analytical techniques available limit what the first 7.5 mA h g^{-1} of charge can be assigned to, but it is likely some form of electrolyte interphase formation that occurs before the first plateau.

The same linear extrapolation method can be used for the emergence of the Mn(III) phase, indicating that Mn oxidation begins at 85 mA h g^{-1}, which is at the start of the second charge plateau. This analysis ascribes 77.5 mA h g^{-1} to Fe oxidation of the PBA, which is half the theoretical capacity. Furthermore, the cathode reaches 84% of the theoretical charge capacity, which agrees well with the 70% phase fraction for Mn(III) at the top of charge, assuming any PBA that is oxidised in the second plateau has already been oxidised in the first. Without wishing to over-interpret this result, these two observations suggest the conversion of capacity to state of charge by this method is physically sensible. The state of charge is measured in units of x in $K_{2-x}Mn[Fe(CN)_6]$ and all further analysis will be in reference to this unit [Fig. S5].

Unexpectedly, there is a region in Fig. S5, early on in the second charge plateau, where all three components are present. This observation is explained by directly interpreting the spectra of component 1 and 2. As soon as component 3 begins to grow in, the linear combination of components 1 and 2 can be assigned as the $KMn^{II}[Fe^{III}(CN)_6]$ phase. To simplify the MMF results, a new MMF fitting process is applied with fixed components. The pristine spectrum is once again used as the first component, the spectrum before the Mn(III) component grows in is fixed as the

second component and the Mn(III) component from the initial ‘free’ MMF refinement is fixed as the third component [Fig. S6]. This new ‘fixed’ MMF results in a fit-to-data of 12.95%, which is still close to the minimum achieved in the free MMF. The fits for selected charge points in the MMF analysis are shown in Fig. S7.

XRD refinement discussion

Rietveld refinement of the powder sample, the pristine electrode, and the pristine cells were made to confirm no changes to the PBA structure had occurred during processes before cell cycling. The unit cell from the powder sample was also used to determine the zero error for each coin cell. A structure table from the Rietveld refinement of the powder sample is provided in Table S1.

Constrained Pawley refinements of the *operando* diffraction measurements were made in the region $5 < 2\theta < 12^\circ$ since the higher angle region is dominated by reflections from other components of the coin cell (stainless steel and aluminium). Unit cells for the other two phases present on cycling were determined by mixed phase Pawley refinements of specific diffraction patterns where they were most intense.

The film plot of the first charge cycle clearly shows the emergence of new phases and disappearance of the pristine phase [Fig. 2C]. The XRD data were normalised by state of charge, and comparison with MMF analysis clearly suggests each phase present in the XRD film plot is closely associated with one of the components from the MMF analysis. First, we focus on the first charge plateau where the pristine monoclinic $K_2Mn[Fe(CN)_6]$ converts to an intermediate phase with Fe oxidation. Pawley refinements of the two phases present showed the intermediate phase is larger in volume than the pristine phase, which at first sight might seem counterintuitive since—based on oxidation of Fe shorting its bonds—one might expect the framework to contract. However the framework in the $K_2Mn[Fe(CN)_6]$ structure is collapsed by octahedral tilts, driven by the K-ion being smaller than the cavity in the cube (*33*). Removal of half of the K-ions unwinds these tilts which expands the framework, increasing the volume by 12%.

The intermediate phase was refined from the pattern collected at $x = 1.06$ by fixing the intensities of the discharged phase and only allowing one phase fraction parameter to refine the whole set of peak intensities. The remaining peaks were captured by a 10.4703(9) Å cubic unit cell in $Fm\bar{3}m$. We note that the intermediate phase $K_1Mn[Fe(CN)_6]$ could also be described by the tetragonal space group $P4/mnc$, which is that of the high-temperature phase for $K_2Mn[Fe(CN)_6]$ (*33*). Our diffraction data here do not have sufficient resolution or signal-to-noise to evidence convincingly any lowering from cubic symmetry.

For the second phase transition to the charged phase, the volume change is more conventional

and shrinks by 7% with oxidation of Mn. The constant voltage hold at 4.3 V pushed the state of charge reached to a maximum of $x = 1.84$. This gave rise to a phase fraction of ~95% for the tetragonal phase, isolated from the diffraction pattern [Fig. S8].

The same fitting procedure was used to find the unit cell for the charged phase which was well-fit by a tetragonal unit cell in $I4/mmm$ with lattice parameters $a = 7.2647(6)$ Å, $c = 10.128(6)$ Å, and a small amount of remaining intermediate phase modelled by a fixed intensity Pawley fit. This space group was chosen because when the Γ_3^+ irrep associated with JT distortions in PBAs is applied to the $Fm\bar{3}m$ space group, the $I4/mmm$ space group is generated.

The phase fractions were refined for each pattern in sequence during charge/discharge cycles to produce the plots in the main text by the following method. Peak-shape and intensity ratios were fixed with a singular scale parameter allowed to refine for each phase. Lattice parameters were allowed to refine provided the phase fraction was above 10%. Phases were removed from the refinement when their phase fraction dropped below 5%. When a phase reformed on discharge it was refitted with new peakshape and intensities before fixing again.

The full charge dependence of the unit-cell parameters for each phase is given in the supplementary tables. Subscript P, I, and C denote discharged, intermediate, and charged phase parameters, respectively.

Kinetic model

Our model consists of 10,000 K-ion sites that may or may not be occupied. This array of K-ion sites were then grouped into domains of 100 K-sites each, corresponding to scattering domains of linear dimension ~ 23 Å, a reasonable coherence length for ~ 0.5 Å X-rays. If the composition of a domain was less than 55% then it was assumed to be cubic, and if it was greater than 55% then it is assumed to be monoclinic.

Each K-ion site (labelled j) was assigned a relative probability of being emptied under subsequent charge

$$p(j) = \exp\{-d[1 - x_{\mathrm{loc}}(j)]\} \tag{S1}$$

that depends on the local composition, $x_{\mathrm{loc}}(j)$ of the domain containing j. For ease $0 \leq x_{\mathrm{loc}} \leq 1$ was used, with the upper bound corresponding to the $K_2Mn[Fe(CN)_6]$ case and the lower bound corresponding to the $K_1Mn[Fe(CN)_6]$ case.

The constant d determines how strongly this probability depends on local composition. In the case that $d = 0$, the probability of K-ion extraction is independent of local composition and the phase boundary is a sharp transition [Fig S10A]. A negative value of d is found to give the (expected) case that K-ion removal is easier in locally depleted regions. The model is best able to reproduce the observed phase behaviour including the delayed formation of the intermediate phase due to the initial process of K-ion removal being more uniform [Fig S10C] using a value of $d = -10$. The influence of the d term alone in its ability to alter the behaviour from equilibrium to the experimentally observed non-equilibrium case shows the significance of the mobilities of K-ion based on their local environment. We include a third case ($d = -4$) to further show the impact of the d term.

An important point worth making here is that, in this model, there is very little change in the populations of most compositions (indeed most of them are close to zero) other than those near $x = 1$ and $x = 0$ [Fig. S10D], which is why the voltage at which this transformation would occur is roughly constant throughout the K-ion removal process, forming a plateau in the charge profile. By ignoring the thermodynamics, this analysis effectively assumes that any compositional relaxation process is slow with respect to the electrochemical K-ion removal, which is why previous studies that measured *ex situ* XRD of partially charged cathodes show the same mixed phases observed in

these *in situ* XRD data (*28, 36*).

One limitation of the model, which is intentionally simplistic in its ability to simulate the experimental results using only one term, it cannot account for inter particle heterogeneities based on non-uniform electrodes. However, our pulse-relaxation experiment in Fig. S9 show that cycling at a faster rate followed by relaxation does not produce different structural behaviour. From this observation we determine that inter particle heterogeneities are not a significant contributor to the structural changes we observe.

Figs. S1–S11

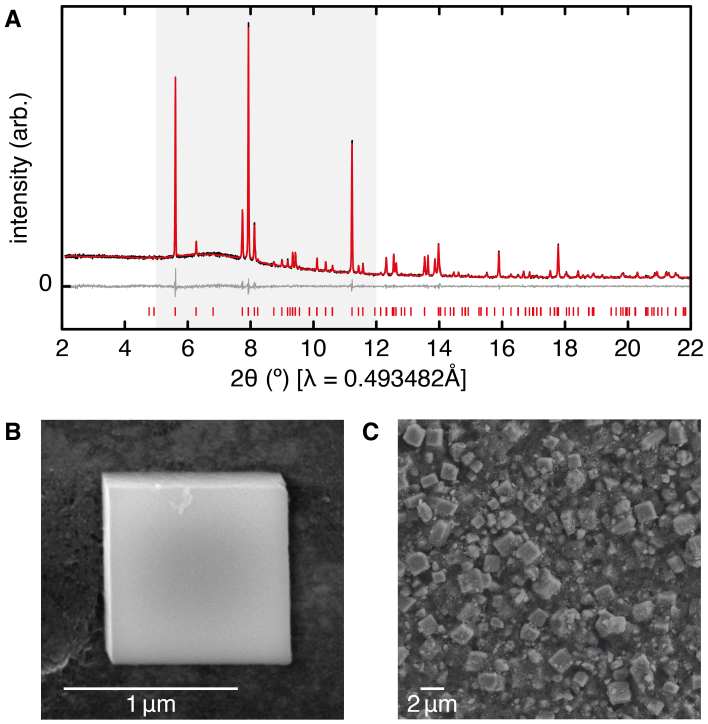

Figure S1: **$K_2Mn[Fe(CN)_6]$ material characterisation.** (A) Rietveld fit for XRD pattern of $K_2Mn[Fe(CN)_6]$ powder sample. Raw data in black, fit in red, difference curve in grey with tickmarks for calculated reflection positions below. The refining region for *operando* XRD is shaded in grey. (B) Scanning electron micrograph of a representative cube of $K_2Mn[Fe(CN)_6]$ and (C) the cathode composite.

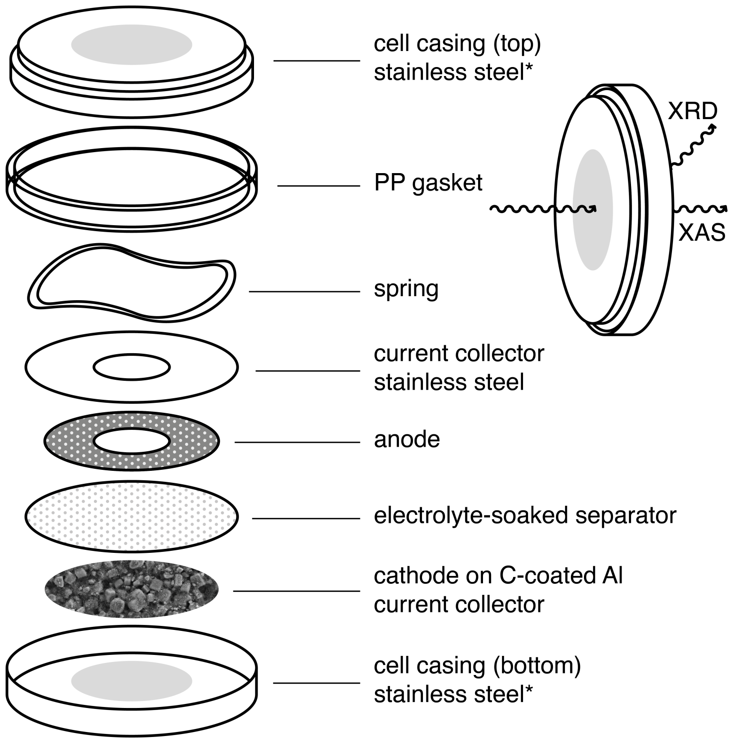

Figure S2: **Modified coin cell for *operando* measurment.** The cell for the *operando* experiments was designed to be as consistent as possible with the conventional coin cell setup. A hole had to be cleared through the stainless steel current collector and K metal anode to allow the beam to pass through. Laser thinning the coin cell casings to 50 μm was sufficient for the 25 keV XRD beam to pass through, but Kapton had to be placed over holes in the casings for the softer (6.5–7.5 keV) X-rays used in the XAS experiment.

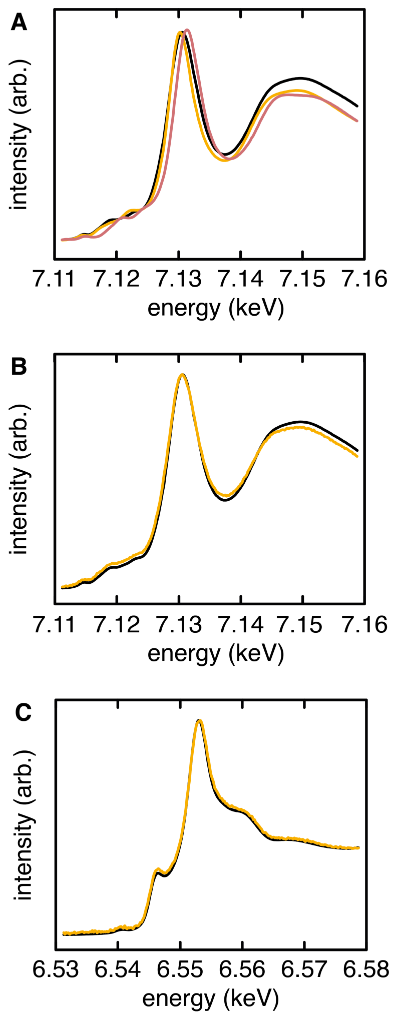

Figure S3: **Reference XAS spectra.** Reference XAS spectra (A) Fe K-edge: $K_4Fe(CN)_6$ (amber) $K_3Fe(CN)_6$ (pink) $K_2Mn[Fe(CN)_6]$ (black). (B) Fe K-edge: pristine cell (amber) $K_2Mn[Fe(CN)_6]$ powder (black). (C) Mn K-dge: pristine cell (amber) $K_2Mn[Fe(CN)_6]$ powder (black).

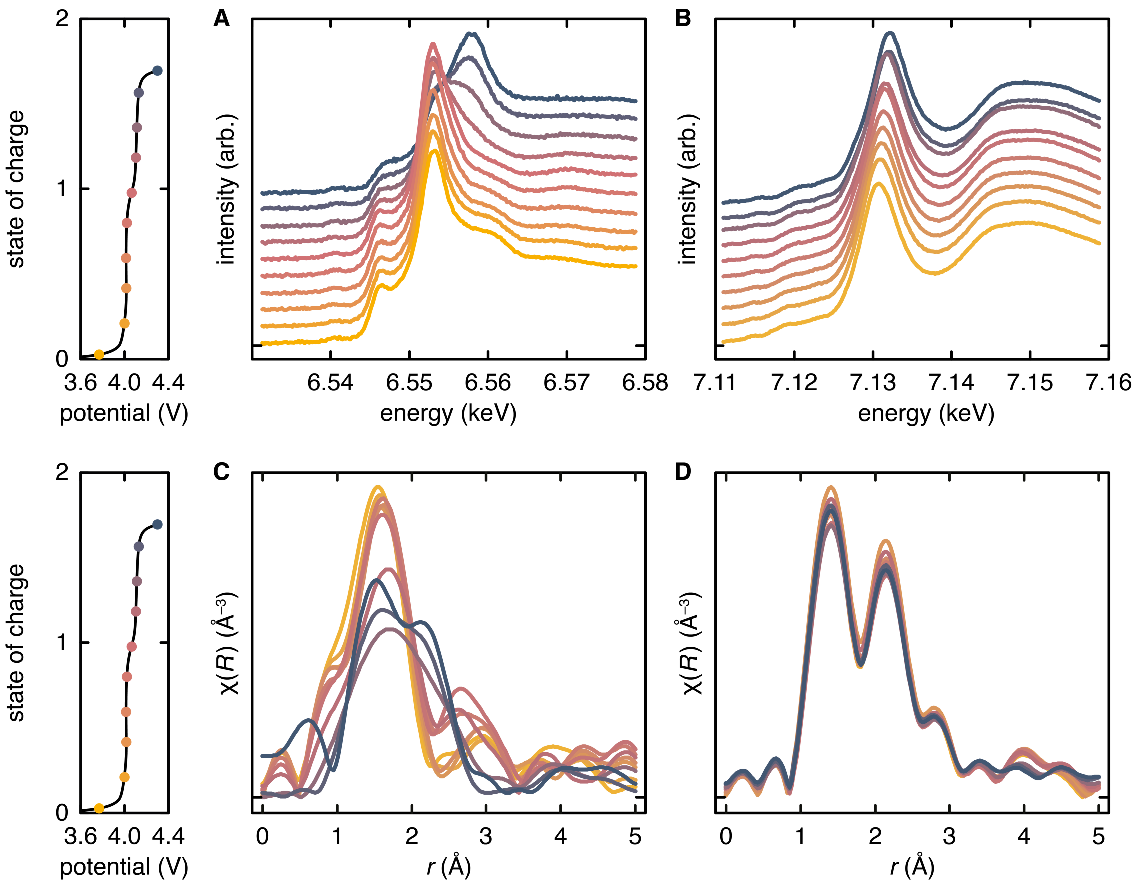

Figure S4: **XAS and EXAFS data.** The normalised XAS profile for the Mn (A) Fe (B) K-edge are plotted with successive curves offset vertically by a constant amount and coloured by state of charge. The EXAFS of Mn (C) and Fe (D) are also plotted with successive curves offset vertically by a constant amount and coloured by state of charge. For the Mn EXAFS a distinct second coordination environment grows in during the second charge plateau, reflecting the assymetric JT-active Mn^{3+}. In contract the Fe EXAFS undergoes very little change due to the buffering effect of the cyanide pi-bond.

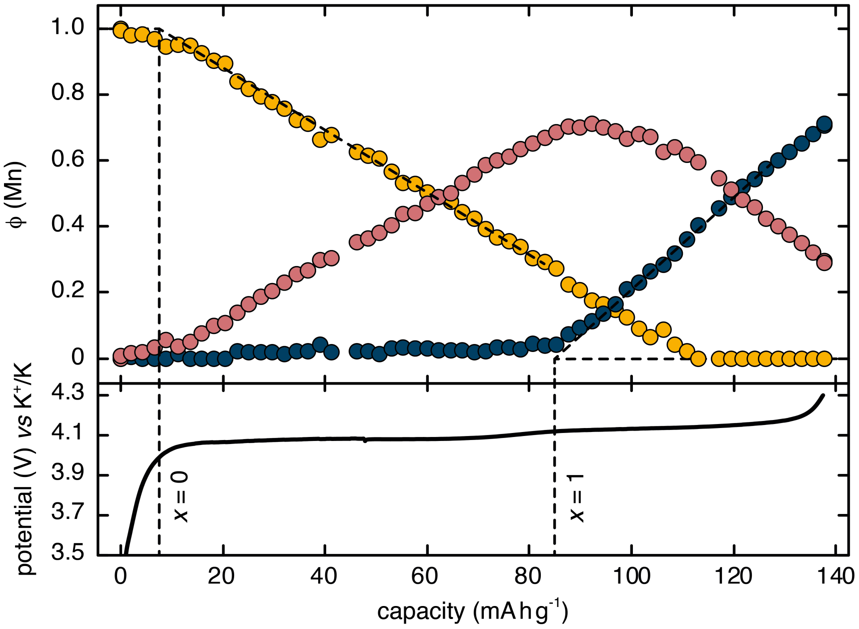

Figure S5: **Process of normalising capacity by state of charge.** Phase fractions, ϕ, for each component from the MMF refinement of the Mn-XAS are plotted against charge capacity. The pristine spectrum is in amber, and the two free components are in pink and dark blue. The linear region of the amber points are extrapolated back to $\phi = 1$ and the linear region of the dark blue points are extrapolated back to $\phi = 0$. The galvanostatic profile of the first charge is plotted to show how the state of charge relates to the charge capacity. In the XAS experiment the cycler had to be restarted at capacity $\simeq$ 48 mA h g^{-1} during the first charge cycle due to a technical fault. The cell was under OCV for 1.5 min and the voltage returned to the plateau after 6 min of charging (0.8 mA h g^{-1}).

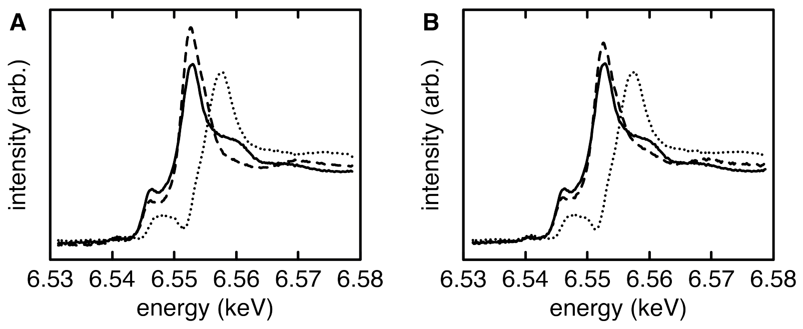

Figure S6: **Component spectra used to fit XAS data by MMF.** (A) For the free MMF refinement, the solid line is the spectrum for the pristine cell, and the dashed and dotted lines are the two refined components (B) In the fixed MMF run the second component is instead fixed as the spectrum at state of charge of 1 (dashed line) and the dotted line is fixed as the third component from the free MMF run.

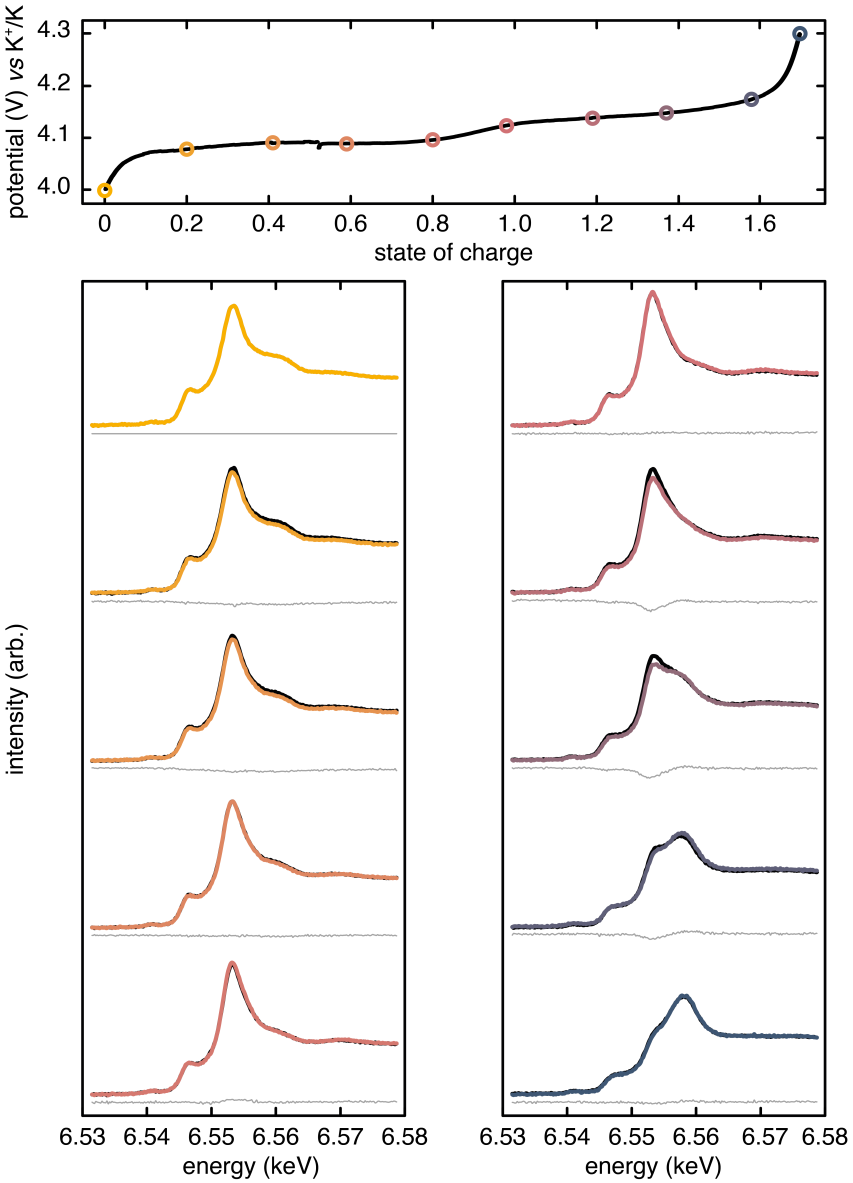

Figure S7: **Fits from the fixed MMF refinement for the spectra in Fig. 2A.** The charge curve above is marked with the points at which spectra were measured. Raw XAS data are plotted in black with the fit overlaid in the colour corresponding to the marker above. A difference curve is plotted below in grey.

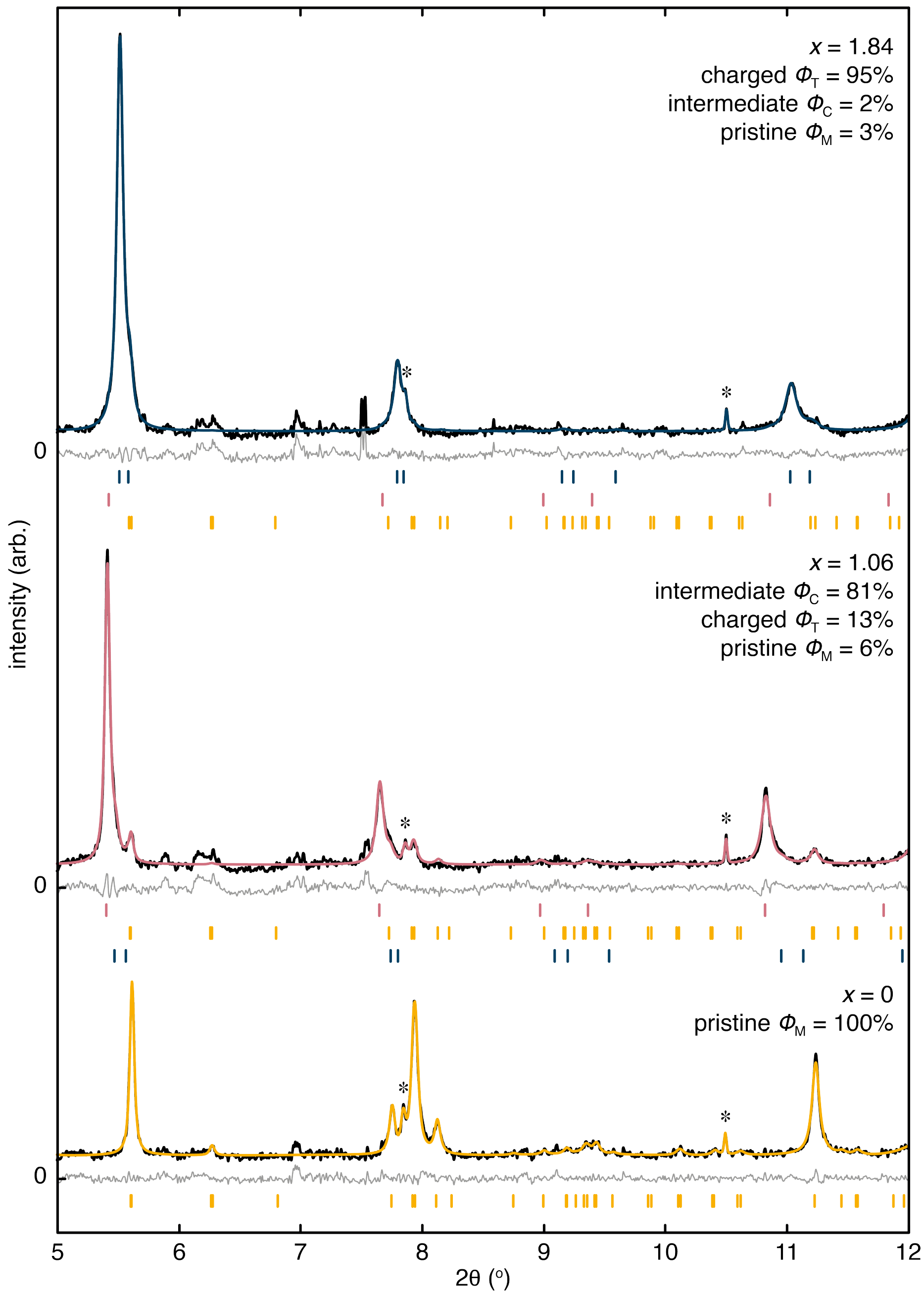

Figure S8: **Pawley fits to the XRD patterns measured for the charged cell (top), the half-charged cell (middle), and the pristine cell (bottom).** Data (black) and fit (coloured) have had the background subtracted, difference function is below in grey, and the calculated reflection positions are shown as coloured tick marks. The dark blue, pink, and amber tick marks correspond to the charged, intermediate, and discharged phases respectively. Asterisks mark reflections contributed by the K metal anode which caught the edge of the beam.

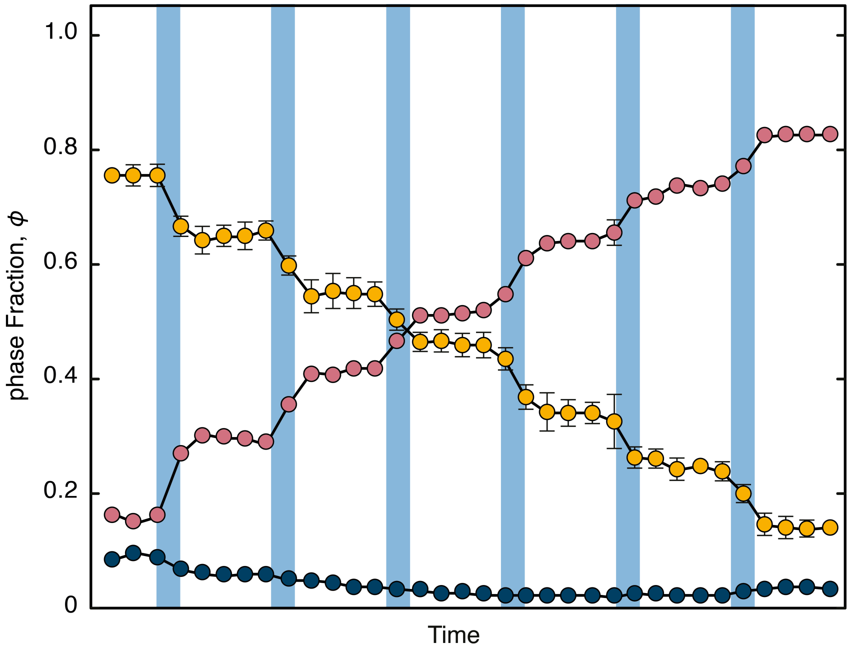

Figure S9: **Pulse-relaxation charging during first charge plateau of second cycle.** The separate phases remain stable when no current is applied and interconvert at a faster rate ($C/10$) in a similar way as the slower cycle in Fig. 3. The time when current is applied is shaded in light blue and any data points without error bars are larger than the uncertainty itself.

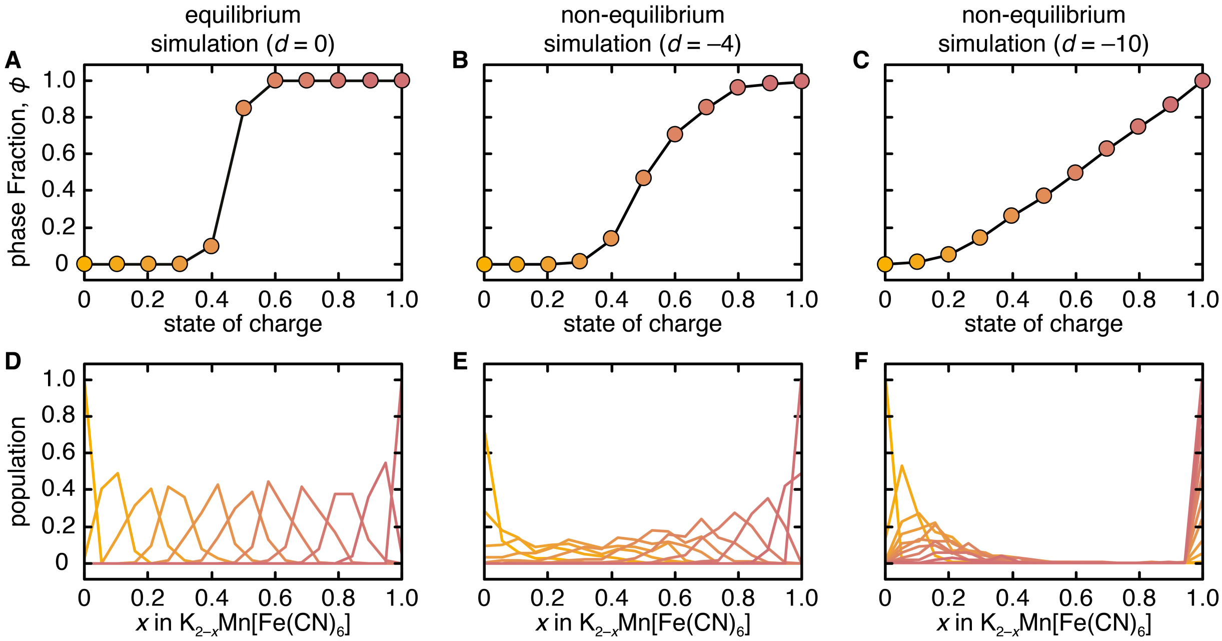

Figure S10: **Kinetic model results.** The three model scenarios are shown by their resultant phase fractions (A,B,C) and their respective $K_{2-x}Mn[Fe(CN)_6]$ distribution (D,E,F). (A) In the first scenario K-ions are extracted at random and the sharp phase transition occurs. (D) Loading, x, in the domains decrease gradually. (B) In the second scenario, K-ion extraction is weighted favour of locally depleted regions, driving a more solid solution behaviour. (E) Domains bias towards high-x or low-x $K_{2-x}Mn[Fe(CN)_6]$. (C) In the third scenario, K-ion extraction is weighted more strongly favour of locally depleted regions, driving a more solid solution behaviour which reflects the observed data. (F) Domains separate into high-x or low-x $K_{2-x}Mn[Fe(CN)_6]$.

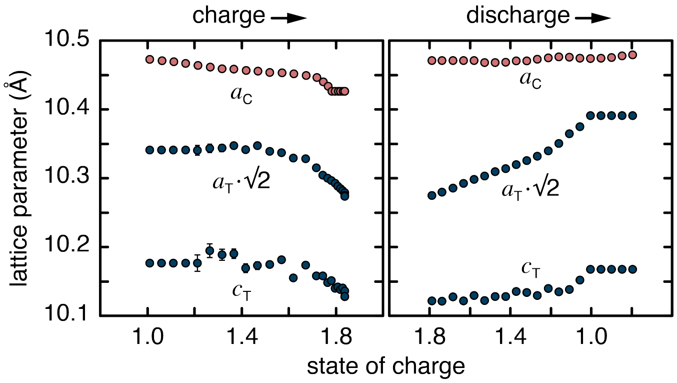

Figure S11: **Charged (T) and intermediate (C) phase lattice parameter variation.** The lattice parameters for the charged and intermediate phases in the high-voltage plateau are extracted from the Pawley refinement and plotted against state of charge with charge and discharge side-by-side. The pink and blue data-points correspond to the intermediate and charged phases, respectively. The a_T lattice parameter from the tetragonal charged phase is converted to a 'pseudo-cubic' cell parameter by multiplying by a factor of $\sqrt{2}$.

Table S1

Table S1: **Crystallographic parameters for the $P2_1/n$ structure of $K_2Mn[Fe(CN)_6]$ at room temperature from the fit the data shown in Fig. S1A.**

a (Å)	10.101(6)			
b (Å)	7.3160(15)			
c (Å)	6.9725(15)			
β (°)	89.954(4)			
V (Å^3)	515.3(3)			
Z	4			
Atom	x	y	z	U_{iso} (Å^2)
Fe	0	0	0	0.0232(9)
Mn	0.5	0	0	0.0232
K1	0.7499(13)	0.5609(4)	−0.0217(6)	0.0452(19)
C1	−0.027(2)	0.853(4)	0.771(4)	0.011(2)
C2	−0.040(2)	0.202(4)	0.838(4)	0.011
C3	0.805(2)	−0.010(2)	0.051(3)	0.011
N1	−0.0435(18)	0.759(3)	0.649(3)	0.011
N2	0.9390(18)	0.334(3)	0.751(3)	0.011
N3	0.699(2)	−0.0503(17)	0.110(3)	0.011

Data S1–S3

Data S1: Phase fraction, lattice parameters, and unit cell volume for the pristine phase modelled by Pawley refinement in $P2_1/n$. Patterns were refined from state of charge 0 to 1.84 with lattice parameters fixed for $x < 1.32$ after-which phase fraction, $\Phi_M < 0.10$. On discharging, patterns were refined in reverse order with lattice parameters fixed for $0.95 < x < 1.79$.

Data S2: Phase fraction, lattice parameters, and unit cell volume for the intermediate phase modelled by Pawley refinement in $Fm\bar{3}m$. Unit cell volume, V_C, is quoted as half the unit cell volume of the cubic unit cell, for consistency with the other two phases. Patterns were refined from state of charge 0 to 1.84 with lattice parameters fixed from $x > 1.81$ afterwhich phase fraction, $\Phi_C < 0.10$. On charging, patterns were refined in reverse order with lattice parameters fixed for $1.63 < x < 1.79$.

Data S3: Phase fraction, lattice parameters, and unit cell volume for the charged phase modelled by Pawley refinement in $I4/mmm$. The phase first appeared on charging at charge state, $x = 1.01$. Lattice parameters were refined until Φ_T dropped below 0.10 on discharging at state of charge, $x = 0.90$.
